# Long‐term of epicardial radiofrequency ablation and benefit for recurrent ventricular arrhythmia in Brugada syndrome: A systematic review and meta‐analysis

**DOI:** 10.1002/joa3.70073

**Published:** 2025-05-08

**Authors:** Arga Setyo Adji, Atiyatum Billah, Juliardi Eka Putra Sit, Bryan Gervais de Liyis, Angga Nugraha, Angela Puspita, Abdillah Maulana Satrioaji, Ragil Nur Rosyadi

**Affiliations:** ^1^ Faculty of Medicine Hang Tuah University Surabaya East Java Indonesia; ^2^ Faculty of Medicine Udayana University Denpasar Bali Indonesia; ^3^ Department of Cardiology, Gadjah Mada University, Yogyakarta, Center Java, Indonesia Yogyakarta Center Java Indonesia; ^4^ Emergency Medicine Division, Department of Internal Medicine Hang Tuah University Surabaya East Java Indonesia; ^5^ Department of Cardiology Rumah Sakit Pusat Angkatan Laut Dr. Ramelan Surabaya East Java Indonesia

**Keywords:** Brugada syndrome, epicardial radiofrequency ablation, ICD shock, long‐term outcomes, ventricular arrhythmia

## Abstract

**Background:**

Brugada syndrome (BrS) is a rare cardiac channelopathy linked to an increased risk of ventricular arrhythmias (VA) and sudden cardiac death. Radiofrequency ablation (RFA), particularly epicardial ablation, is recommended for BrS patients with recurrent VA unresponsive to conventional treatments like implantable cardioverter‐defibrillators (ICD) and quinidine. This study aims to evaluate the long‐term efficacy of epicardial RFA in preventing VA recurrence in BrS.

**Methods:**

A systematic search of PubMed, ScienceDirect, Cochrane Library, and ProQuest databases was conducted following PRISMA 2020 guidelines. Studies on RFA for VA in BrS were included. Primary outcomes were VA recurrence and all‐cause mortality. Statistical analysis was performed using Review Manager 5.4.

**Results:**

Epicardial, endocardial, and combined ablation strategies effectively reduced VA recurrence, decreased ICD shocks, and improved clinical outcomes in BrS. Epicardial ablation RFA near coronary arteries showed a notable reduction in VA recurrence, while endocardial ablation remained a viable alternative. Meta‐analysis revealed a significant reduction in VA recurrence (RR 0.17; 95% CI 0.07–0.43; *p* < .0001) and ICD shocks (RR 0.13; 95% CI 0.04–0.44; *p* = .001). Subgroup analysis suggested greater VA reduction with epicardial ablation, though without statistical significance.

**Conclusion:**

Epicardial RFA is associated with a significant reduction in recurrent VAs (83%) and ICD shock rates (87%) in patients with BrS. The procedure demonstrates a favorable long‐term safety profile, with no mortality reported in the included studies.

## Graphical abstract

Epicardial radiofrequency ablation significantly reduces recurrent ventricular arrhythmias and ICD shocks in Brugada syndrome, with excellent long‐term safety and no reported mortality. Epicardial ablation is an effective strategy for sustained rhythm stabilization.

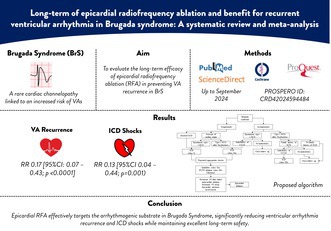

## 1 INTRODUCTION

Brugada syndrome (BrS) is a rare electrical abnormality of the heart.^1^ BrS is relatively uncommon, with an estimated global prevalence of 0.05% and a Brugada pattern frequency of 0.4%. However, individuals of Southeast Asian descent show a higher prevalence, at approximately 3.7 per 1000.^2^ Patients who initially arrived with syncope and a spontaneous BrS ECG sign had a 19% likelihood of recurrence, compared to 69% for those who present with aborted sudden death.^3^ Because of the underlying genetic and cellular anomalies in the heart's electrical circuitry, ventricular arrhythmia (VA) in BrS may recur.

Quinidine or catheter ablation has been effective in lowering the frequency of implantable cardioverter‐defibrillator (ICD) shocks in patients with ventricular fibrillation (VF) who experience recurrent shocks. However, up to 37% of people may experience negative side effects from quinidine, and quinidine is unavailable in many nations. The ST‐segment elevation in the right precordial leads and the incidence of VF in BrS patients may be caused by an aberrant fibrotic arrhythmogenic substrate in the epicardial Right Ventricular Outflow Tract (RVOT), according to electrophysiological mapping data connected to histological investigations. In 75% of patients, ablation of these aberrant regions can significantly reduce recurrent VF and return the ECG to normal.^4^, ^5^

The main cause of BrS is an aberrant electrical substrate in the right ventricular outflow tract (RVOT), more precisely in the myocardium's epicardial layer. Abnormalities in depolarization and repolarization cause reentrant circuits, delayed activation, and slowing of conduction, which increases the risk of VF or ventricular tachycardia (VT). The distinctive ST‐segment elevation in BrS is caused by epicardial anomalies, including conduction delay and decreased sodium channel expression.^6^

In BrS, the arrhythmogenic substrate is frequently limited to the RVOT's epicardial surface. Arrhythmia‐causing triggers and circuits are eliminated by ablation of this epicardial substrate.^7^ Endocardial ablation is inadequate in BrS because the aberrant substrate is mainly in the epicardium. In drug‐resistant VF, endocardial ablation of VF triggers with substrate modification is a reasonable first line of treatment. If QRS notch or fractionated electrograms are present at the endocardial surface of the RVOT, endocardial ablation is insufficient, and an epicardial approach may be a more successful final strategy. This study aimed to evaluate the long‐term benefits of epicardial RFA and its benefit for recurrent VA in BrS.

## 2 METHODS

### 2.1 Study design

A thorough search strategy was implemented to identify relevant studies for inclusion in this systematic review and meta‐analysis. Searches were conducted across several electronic databases, including PubMed, Science Direct, Cochrane Library, and ProQuest. The database for BrS ablation studies was first accessed on June 2024. The search spanned from the inception of each database up to September 2024. The keywords used for the search included “ablation,” “brugada,” and “ventricular arrhythmia.” Titles and abstracts of all identified studies were screened, and reference lists from these studies were also reviewed to locate additional relevant research. The search process followed PRISMA 2020 guidelines, and the study was prospectively registered in the PROSPERO database (CRD42024594484). Study selection was carried out independently by the authors, who applied predefined inclusion criteria, focusing on studies investigating outcomes of catheter or surgical RFA in patients with BrS, using previously established diagnostic criteria. Final study eligibility was determined after a full‐text review, and any disagreements were resolved through discussion among the authors.

### 2.2 Eligibility criteria

Studies were eligible for inclusion if they met the following criteria: (1) the study population comprised patients diagnosed with BrS according to established diagnostic criteria; (2) the intervention involved catheter‐based or surgical RFA aimed at treating VA in BrS; (3) the study reported relevant clinical outcomes, including VA recurrence and/or all‐cause mortality; and (4) both interventional and observational study designs were considered. No restrictions were applied with regard to language, publication year, or duration of follow‐up.

### 2.3 Outcomes measured

The primary outcomes assessed in this study include the recurrence of VT, sudden cardiac death (SCD), and all‐cause mortality. Any additional relevant outcomes were incorporated if present. VT recurrence was defined by the documentation of sustained episodes of VT during follow‐up, identified through ECG, 24‐hour Holter monitoring, or appropriate ICD responses (shock or anti‐tachycardia pacing). VT recurrence data were gathered pre‐ and postablation for each patient. SCD was defined as unexpected death from cardiac causes occurring suddenly, usually within 1 h, as documented during follow‐up. All‐cause mortality included any death reported during the follow‐up period, regardless of cause. Subgroup analyses were performed based on the ablation site, whether epicardial, endocardial, or a combination of both.

### 2.4 Data extractions and quality appraisal

Standardized forms were used to extract key information from each included study, covering three main areas: study characteristics, population characteristics, and outcomes. For study characteristics, the following details were recorded: (i) author and year of publication, (ii) country of study, (iii) study design and methodology, (iv) total sample size, and (v) follow‐up duration. Population characteristics included: (i) baseline demographic and clinical features, (ii) type of ablation procedure (catheter and/or surgical), and (iii) ablation site (epicardial and/or endocardial). Outcome data were extracted based on the endpoint measures specified in the study, with a focus on the primary and secondary outcomes outlined in the current meta‐analysis protocol.

The methodological quality of the studies will be assessed using appropriate evaluation tools. For RCTs, the assessment will be conducted using the Cochrane Risk of Bias Tool, which evaluates bias across seven domains, including randomization, allocation concealment, blinding of participants and researchers, and outcome reporting. For nonrandomized studies, quality assessment will be conducted using the Newcastle‐Ottawa Scale (NOS), which evaluates participant selection, group comparability, and outcome assessment. Potential biases, whether from study design, reporting, or publication, will be identified and reported. Any discrepancies between reviewers during the quality assessment were resolved through detailed discussions. If consensus could not be reached, a third independent reviewer was consulted to adjudicate the differences.

### 2.5 Quality assessment and statistical analysis

For interventional studies, such as randomized controlled trials (RCTs), the risk of bias was categorized as low, moderate, or high. Observational studies were assessed using the previously stated scoring system, classifying them as low quality (<5 points), moderate quality (5–7 points), or high quality (>7 points). Only studies with low risk of bias or rated as high quality were included in the quantitative analysis.

Data for a specific variable were synthesized only if it was reported in at least two included studies. Continuous variables were presented as means with standard deviation (SD). Heterogeneity among study populations was evaluated using the *I* ^2^ and *T* ^2^ statistics. When *I* ^2^ was <50% or *T* ^2^ >0,05, a Mantel–Haenszel (M‐H) fixed‐effect model was applied to summarize data; if criteria were not met, a random‐effects model was used. Subgroup analyses of primary outcomes were compared with the *Z* statistic. Funnel plots were used to assess publication bias. All analysis was performed using Review Manager 5.4.

## 3 RESULTS

### 3.1 Selection of the studies

**FIGURE 1:**
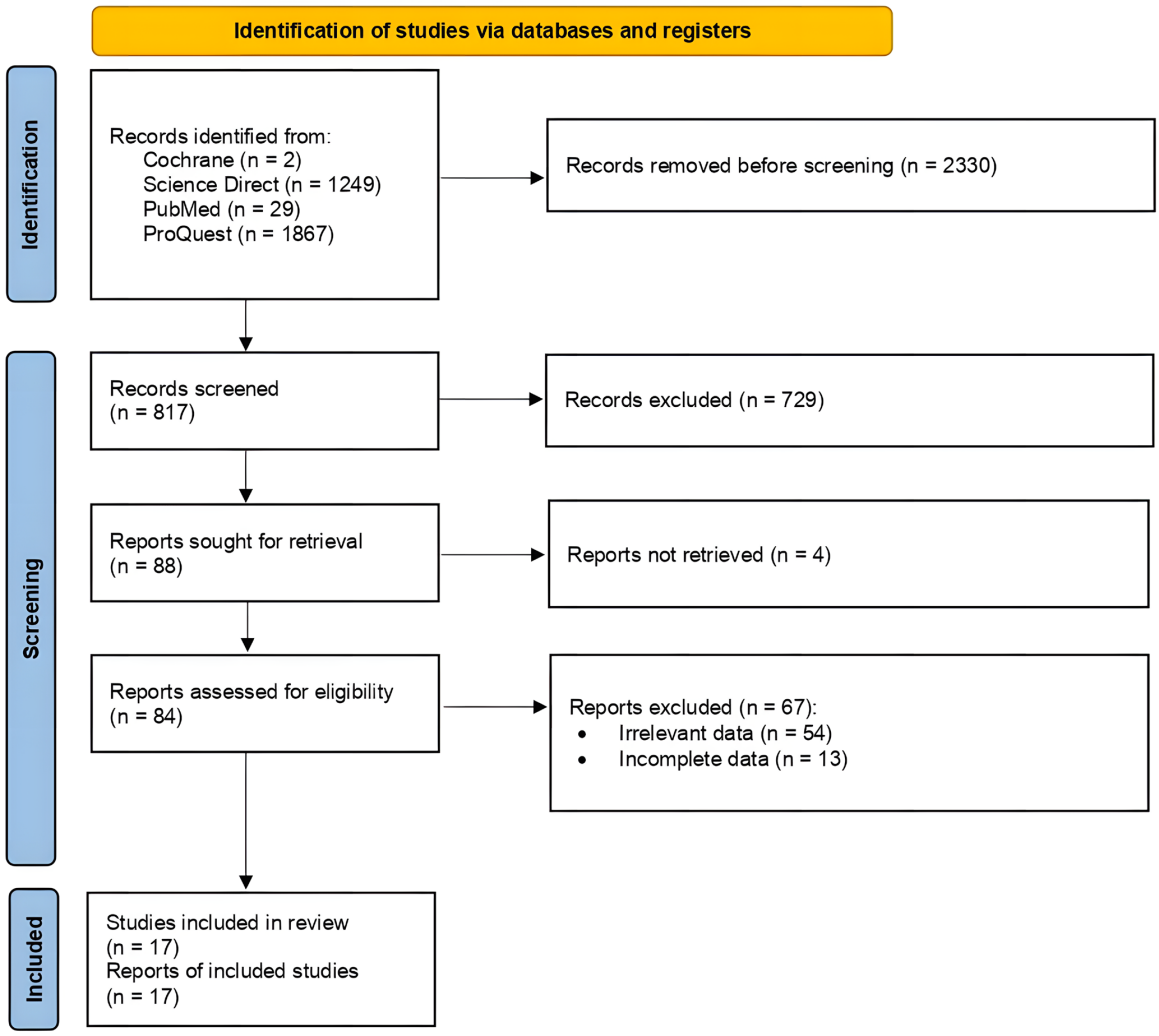
PRISMA flow diagram.

The selection of study process adhered to the PRISMA 2020 guidelines. At the beginning of the study selection process, a primary search was conducted, which yielded an initial total of 3147 studies. After following the PRISMA guideline, a total of 17 studies met all the inclusion criteria and were suitable to include in this study (Figure 1).

### 3.2 Characteristics of included studies

There was a total of 17 studies included in this study.^4^, ^8^, ^9^, ^10^, ^11^, ^12^, ^13^, ^14^, ^15^, ^16^, ^17^, ^18^, ^19^, ^20^, ^21^, ^22^, ^23^ All of the included studies were cohort studies. The population of the included studies was 769 patients in total, with 712 BrS patients. Most of the included studies were conducted in Asia. The shortest follow‐up duration of the included studies was approximately 5 months,^8^ while the longest was 86.4 months.^15^ The ablation techniques used in the included studies were epicardial ablation, endocardial ablation, and the combination of those two techniques. The outcomes measured in the included studies included ECG pattern and parameter,^4^, ^8^, ^9^, ^10^, ^11^, ^13^, ^14^, ^15^ VA recurrence,^4^, ^8^, ^9^, ^11^, ^12^, ^13^, ^14^, ^15^, ^16^, ^17^, ^18^, ^19^, ^20^, ^22^, ^23^ complication rate,^8^, ^9^, ^11^ SCD recurrence,^20^ and ICD shock.^9^, ^11^, ^21^ From the included studies, ablation treatment either—epicardial, endocardial, or a combination of both—is effective in preventing VA recurrence, decreasing the number of ICD shocks in BrS, and improving the arrhythmic outcomes in patients who decline ICD implantation. Although not all of the included studies performed endocardial ablation, some of them reported that endocardial ablation can be considered an alternative to epicardial ablation when targeting regions near the coronary artery^12^, ^15^, ^19^, ^20^, ^22^ (Table 1).

**TABLE 1:** Characteristics of included studies.

No.	Author, year	Study design	Country	Total population	Follow up duration (months)	Type of ablation catheter	Ablation technique	Ablation procedure	Outcomes measured	Main results
1	Brugada, 2015^8^	Cohort	Spain	14	5 months (3.8–5.3 months)	An externally irrigated 3.5‐mm tip ablation catheter (ThermoCool SF, Navistar, Biosense Webster).	Epicardial ablation	The reported ablation time was a median of 23.8 min (range: 18.1–28.5 min). The study noted successful substrate elimination with most patients requiring only a single procedure, with a small number needing repeat ablation.	Elimination of BrS ECG pattern, VT recurrence, complication rate	Low voltage areas increased from 17.6 cm^2^ to 28.5 cm^2^ after flecainide administration (*p* = 0.001). Abnormal electrograms increased from 19.0 cm^2^ to 27.3 cm^2^ after flecainide administration (*p* = 0.001). After 23.8 min of RFA, abnormal electrograms disappeared and low‐voltage areas were replaced by scar tissue (<0.5 mV). No complications were reported.
2	Chung, 2017^16^	Cohort	Taiwan	15	18.2 ± 9.0 months	A multipolar mapping catheter	Epicardial ablation	Continous irrigation at a rate of 2 mL/min, mean RF application duration of 27.5 ± 10.5 min	VA recurrence	Targeting triggers and abnormal epicardial substrates through ablation has proven to be an effective approach for preventing recurrences of ventricular tachyarrhythmias in BrS.
3	Haïssaguerre, 2003^17^	Cohort	France	3	17 ± 17 months	A 4‐mm‐tip quadripolar roving ablation catheter equipped with a thermocouple (Biosense Webster)	Epicardial ablation	Acute elimination of premature beats was achieved after approximately 12 ± 6 min of radiofrequency application.	VA recurrence, complication rate	No recurrence of symptomatic VA during a mean follow‐up of 17 months. One patient had persistent premature beats but no arrhythmia recurrence.
4	Haïssaguerre, 2022^18^	Cohort	France	54	56 ± 30 months	NR	Catheter ablation	NR	VF recurrence	Sixty‐one episodes of VF (10 spontaneous and 51 induced) were charted, with. In BrS cases, the ablation used targeted the right ventricle and structural substrate resulting in a reduction in VF recurrence from a median of 7 episodes pre‐procedure (IQR 4–16) to 0 episodes (IQR 0–2) at follow‐up.
5	Kamakura, 2021^19^	Cohort	Japan	16	25.1 ± 29.1 months	A 3.5‐mm, open‐irrigated catheter (NAVISTAR or ThermoCool, Biosense Webster)	Endocardial and epicardial ablation	Using a power of 25–35 W for the epicardium and 20–50 W (mostly 30–40 W) for the endocardium.	VA recurrence	Endocardial ablation could be recommended as an alternate to epicardial ablation for treating areas near the coronary artery.
6	Li, 2024^20^	Cohort	China	18	73.7 months	A 3.5‐mm‐tip NaviStar‐ThermoCool catheter (Biosense Webster, Diamond Bar, CA)	Endocardial and epicardial ablation	NR	ECG parameters, VF recurrence, SCD recurrence	Patients with symptomatic BrS who refuse ICD placement can improve their arrhythmic outcomes with catheter ablation, an effective alternative treatment.
7	Mamiya, 2021^21^	Cohort	Japan	11	42 months	An irrigated 3.5‐mm tip ablation catheter (NaviStar ThermoCool; BiosenseWebster) with a power setting of 30 W	Epicardial ablation	The mean total ablation energy applied to the area and ablation time were 80,705 ± 16,006 J and 40.4 ± 10.3 min	ECG parameters, ICD shock	The number of ICD shocks was significantly decreased by catheter ablation for epicardial arrhythmogenic substrate in BrS, with potential modifications in ECG parameters.
8	Manero, 2015^22^	Cohort	Spain	6	69.4 ± 54.3 months	NR	Endocardial and epicardial ablation	NR	VA recurrence	Endocardial ablation (in one case combined with epicardial ablation) effectively controlled symptoms during short‐term follow‐up.
9	Nademanee, 2019^23^	Cohort	Thailand	52	31 ± 26 months	A high‐density endocardial and epicardial mapping with the CARTO system.	Catheter ablation	For patients undergoing VF substrate ablation (with or without additional trigger ablation), the mean number of sessions was 1.4 ± 0.6. In patients treated exclusively with Purkinje trigger ablation, the mean was 1.2 ± 0.4.	Localization of VF substrates and triggers through mapping, reduction in VF recurrences after ablation	Forty patients exhibited late depolarization abnormalities, primarily at the right ventricular (RV) epicardium, with 33 of these patients showing a Brugada electrocardiographic pattern.
10	Nademanee, 2023^9^	Cohort	Thailand	159	28.29 months	CARTO (Biosense Webster) or Ensite Precision (Abbott)	Epicardial ablation	Radiofrequency power was titrated between 20 and 50 W depending on the contact force. The radiofrequency duration time was 25 ± 16 min (median, 22 min).	ECG parameters, VF recurrence, ICD shock, periprocedural complications	For high‐risk BrS patients, ablation therapy is a safe and very effective way to stop VF recurrence. A serious periprocedural complication occurred in 4 patients, who developed hemopericardium
11	Pappone, 2017^10^	Cohort	Italy	135	10 months (3–13 months)	An externally irrigated 3.5‐mm tip ablation catheter (ThermoCool SF, Navistar, Biosense Webster).	Radiofrequency ablation	An externally irrigated 3.5‐mm tip ablation catheter (ThermoCool SF, Navistar, Biosense Webster). The ablation was completed in a median time of 23.8 min (range: 18.1–28.5 min). The majority of patients underwent a single procedure; only 2 patients required a repeat ablation for recurrent ventricular fibrillation.	AES, ECG pattern normalization and noninducibility of VT and VF	Radiofrequency ablation successfully eliminated AES in all patients, leading to ECG normalization and noninducibility of VT/VF. All but 2 patients maintained a normal ECG postprocedure, even after ajmaline, with the two requiring a repeat procedure for recurrent VF.
12	Salghetti, 2019^11^	Cohort	Belgia	36	30 months	A bipolar unidirectional RF linear device (Coolrail, AtriCure Inc., West Chester, OH) or with a bipolar 3.5‐mm tip ablation catheter (ThermoCool SF, Navistar, Biosense Webster; FlexAbility, St. Jude Medical), set without irrigation	Epicardial ablation	Total procedure time 42 ± 3 min, Ablation time 12 ± 5 min	Electrogram, ECG parameters, VF recurrence, complications, ICD shock	Hybrid thoracoscopic epicardial ablation of the RVOT is a new, safe, and practical technique for identifying and eliminating pathological substrate areas in BrS, offering direct control over the RF ablation process.
13	Santinelli, 2024^4^	Cohort	Italy	206	24 months	A dedicated high‐density mapping catheter (DECANAV catheter, Biosense Webster)	Epicardial ablation	NR	Risk factors for VF events before ablation, ECG parameters, VF recurrence	When paired with ICD implantation, epicardial ablation provides a proven and efficient way to improve outcomes over the no‐RFA group.
14	Shelke, 2018^12^	Cohort	India	5	46 months (4–81 months)	An 8F deflectable CELSIUS Thermocool D curve catheter (Biosense Webster, Diamond Bar, CA, USA), or a 7F deflectable Therapy Coolpath medium curve catheter	Endocardial and epicardial ablation	Ablation performed in sinus rhythm, targeting fractionated potentials, split EGMs, isolated late potentials (ILP), and low‐voltage areas (<1.5 mV), RFA deliver 30 W energy at 43 C catheter tip temperature at an irrigation rate of 2 mL/min in the epicardium and up to 30 mL/min in the endocardium.	Shock, VA recurrence	Substrate modification in patients with RVOT in BrS may eliminate Brugada pattern on the ECG and avoids spontaneous VA on long‐term follow‐up.
15	Talib, 2018^13^	Cohort	Japan	21	55 months	An externally irrigated 3.5‐mm‐tip catheter (Thermocool, Biosense Webster, Diamond Bar, CA)	Epicardial ablation	Total radiofrequency time was 29 ± 11 min for QRS notch in lead V1 and 26 ± 8 min for no QRS notching	ECG parameters, VF recurrence	The long‐term result of eliminating endocardial VF trigger and then modifying endocardial substrate is great.
16	Tokioka, 2020^15^	Cohort	Japan	7	86.4 months	A 3.5‐mm‐tip irrigated catheter, Biosense Webster, CA) or THERMOCOOL®SF (3.5‐mm‐tip irrigated catheter, Biosense Webster, CA)	Endocardial ablation	NR	ECG parameters, VF recurrence	By using catheter ablation, all instances of monomorphic VT were effectively treated.
17	Zhang, 2016^14^	Cohort	China	11	42 months	A 3.5‐mm‐tip NaviStar‐ThermoCool catheter (Biosense Webster, Diamond Bar, CA)	Epicardial ablation	NR	ECG parameters, VF recurrence	Recurrence was noted even after ablation, when all patients' spontaneous type 1 patterns returned to normal, indicating that ICD should be the primary treatment for BrS.

### 3.3 Quality of included studies

The NOS scale examines several key factors that influence the overall reliability of a study, including selection, comparability of cohorts, and the assessment of outcome. Each of these criteria is scored, allowing researchers to rate the study quality on a spectrum ranging from poor to good. Based on the NOS for cohort studies, the studies included had fair to good quality (Table 2).

**TABLE 2:** Quality of the included studies.

Author, year	Selection domain	Comparability domain	Outcome domain	Total score	Study quality
Brugada, 2015^8^	☆ ☆ ☆	☆ ☆	☆ ☆	7	Good
Chung, 2017^16^	☆ ☆ ☆	☆ ☆	☆ ☆	7	Good
Haïssaguerre, 2003^17^	☆ ☆ ☆	☆	☆ ☆	6	Good
Haïssaguerre, 2022^18^	☆ ☆ ☆	☆ ☆	☆ ☆ ☆	8	Good
Kamakura, 2021^19^	☆ ☆ ☆	☆ ☆	☆ ☆	7	Good
Li, 2024^20^	☆ ☆ ☆	☆ ☆	☆ ☆ ☆	8	Good
Mamiya, 2021^21^	☆ ☆ ☆	☆	☆ ☆	6	Good
Manero, 2015^22^	☆ ☆ ☆	☆ ☆	☆ ☆ ☆	8	Good
Nademanee, 2019^23^	☆ ☆ ☆	☆ ☆	☆ ☆	7	Good
Nademanee, 2023^9^	☆ ☆ ☆	☆ ☆	☆ ☆	7	Good
Papone, 2017	☆ ☆ ☆ ☆	☆ ☆	☆ ☆ ☆	9	Good
Salghetti, 2018	☆ ☆ ☆	☆ ☆	☆ ☆ ☆	8	Good
Santinelli, 2024^4^	☆ ☆ ☆	☆ ☆	☆ ☆ ☆	8	Good
Shelke, 2018^12^	☆ ☆ ☆	☆ ☆	☆ ☆	7	Good
Talib, 2018^13^	☆ ☆ ☆	☆	☆ ☆ ☆	7	Good
Tokioka, 2020^15^	☆ ☆	☆	☆ ☆	5	Fair
Zhang, 2016^14^	☆ ☆ ☆	☆	☆ ☆	6	Good

### 3.4 Recurrent ventricular arrhythmia

**FIGURE 2:**
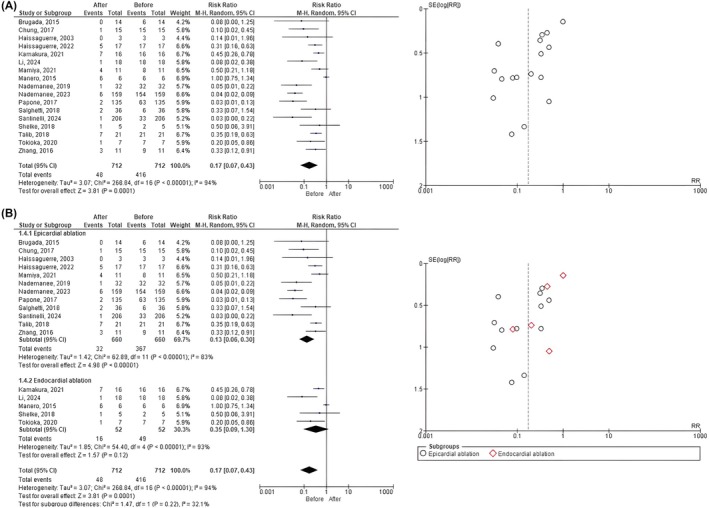
Forest plot analysis of (A) overall recurrent VA event before and after ablation; and (B) subgroup analysis based on ablation site.

Our analysis included a total of 712 patients with BrS, shows that RFA is associated with a substantial reduction in event recurrence in patients, as indicated by the pooled risk ratio of 0.17 (CI95% 0.07–0.43; *p*‐value <.0001). However, the studies included show a high level of heterogeneity, indicated by an *I* ^2^ value of 94% (*p*‐value <.00001) suggesting differences in the magnitude of the effect across the studies. Furthermore, the funnel plot indicates some degree of asymmetry, which may suggest the presence of publication bias or heterogeneity, especially among the smaller studies (Figure 2A).

To address this issue and gain further insight into the differential effects of the ablation strategies, we performed subgroup analyses based on the ablation site—specifically, epicardial versus endocardial ablation. This stratification was chosen because Brugada syndrome is characterized by an arrhythmogenic substrate predominantly located in the epicardium. Epicardial ablation, therefore, represents a targeted substrate modification approach, whereas endocardial ablation primarily aims to eliminate arrhythmia triggers, such as premature ventricular contractions and monomorphic ventricular tachycardia. By analyzing these subgroups separately, we sought to clarify the relative effectiveness of each technique and provide a more nuanced interpretation of the treatment outcomes.

In subgroup analysis, the overall pooled risk ratio for epicardial ablation and endocardial ablation was 0.13 (95% CI 0.06–0.30; *p*‐value <.00001) and 0.35 (95% CI 0.09–1.30; *p*‐value = .12), which suggests a significant reduction in the event rates after epicardial ablation but not after endocardial ablation. However, the test for subgroup differences showed that there was no statistically significant difference between the epicardial and endocardial subgroups (Chi^2^ = 1.47; *p*‐value = .22; *I* ^2^ = 31.1%). Furthermore, the funnel plot also indicates some degree of asymmetry both in the epicardial and endocardial ablation groups, which may suggest the presence of publication bias or heterogeneity, especially among the smaller studies (Figure 2B).

### 3.5 Implantable cardioverter‐defibrillator shocks

**FIGURE 3:**
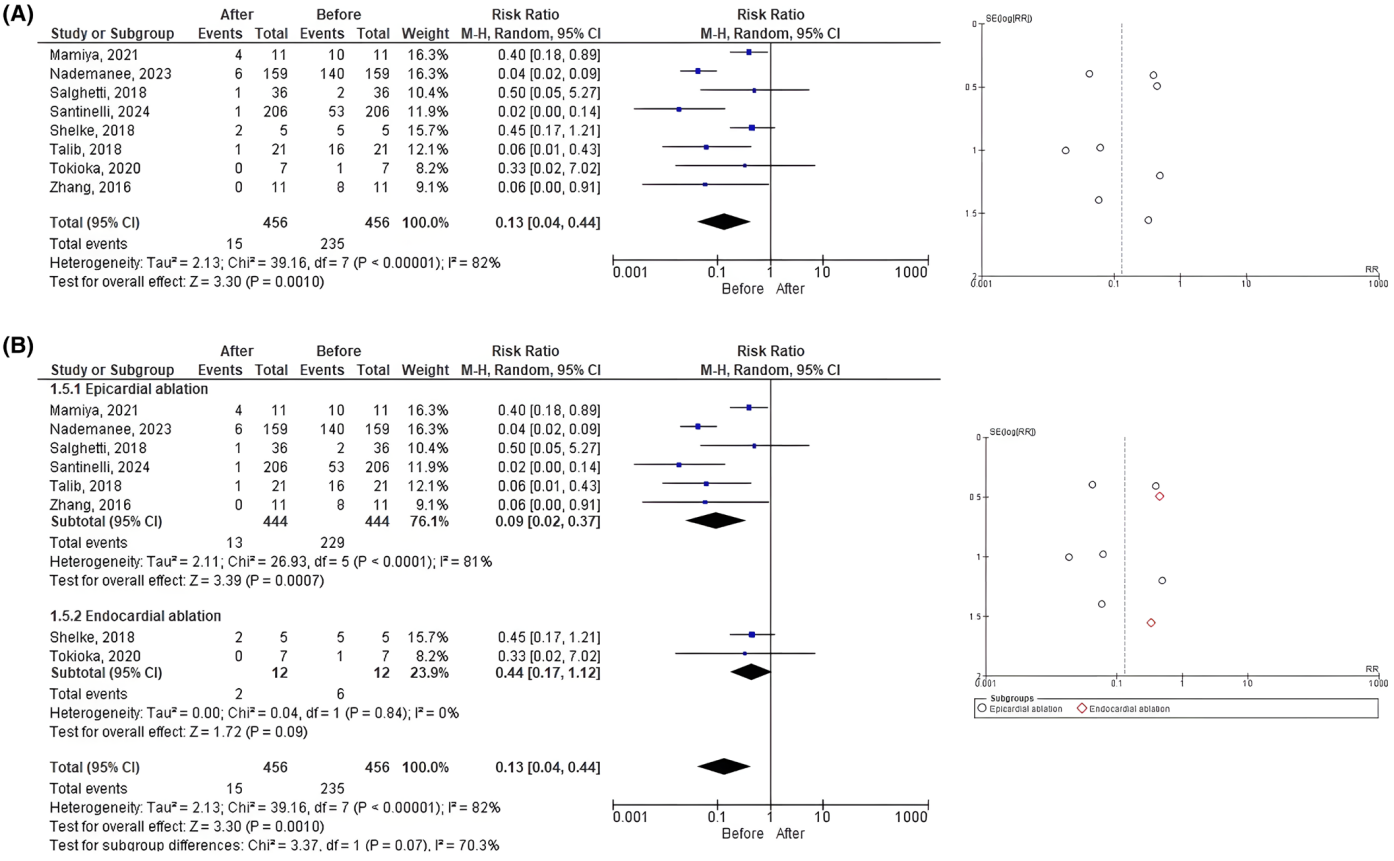
Forest plot analysis of (A) implantable cardioverter‐defibrillator shocks event before and after ablation; and (B) subgroup analysis based on ablation site.

Our analysis included a total of 456 patients with BrS, showing that RFA is associated with a substantial reduction in ICD shock in patients, as indicated by the pooled risk ratio of 0.13 (CI 95% 0.04–0.44; *p*‐value = .0010). However, the studies included show a high level of heterogeneity, indicated by an *I* ^2^ value of 82% (*p*‐value <.00001) suggesting differences in the magnitude of the effect across the studies. Furthermore, the funnel plot indicates some degree of asymmetry, which may suggest the presence of publication bias or heterogeneity, especially among the smaller studies (Figure 3A).

In subgroup analysis, the overall pooled RR for epicardial ablation and endocardial ablation was 0.09 (95% CI 0.02–0.37; *p*‐value = .0007) and 0.44 (95% CI 0.17–1.12; *p*‐value = .09), which suggests a significant reduction in the event rates after epicardial ablation but not after endocardial ablation. However, the test for subgroup differences showed that there was no statistically significant difference between the epicardial and endocardial subgroups (χ^2^ = 3.37; *p*‐value = .07; *I* ^2^ = 70.3%). Furthermore, the funnel plot also indicates some degree of asymmetry both in the epicardial and endocardial ablation groups, which may suggest the presence of publication bias or heterogeneity, especially among the smaller studies (Figure 3B).

### 3.6 Complications

Brugada et al. reported no complications. Nademanee et al. reported a serious periprocedural complication that occurred in four patients, who developed hemopericardium. Haïssaguerre et al. reported nine patients had one or two VF recurrences, and seven patients had more than two recurrences. Salghetti et al. reported one patient developed a left pneumothorax the day after the procedure, which was managed by pleural drainage. Nine patients developed pericarditis between 2 and 10 days after ablation. One patient experienced hematic cardiac tamponade. Li et al. reported that in the ICD group, 12 patients had recurrent VF episodes, resulting in multiple ICD shocks, and among them, two patients also experienced inappropriate ICD shocks. Manero et al. showed two patients had RVOT‐VT either during or after epicardial VT ablation, and in other cases, endocardial ablation failed three times to abolish VT‐VPB. Pappone et al. recorded one case of VT and one case of VF.

### 3.7 All‐cause mortality

None of the included studies reported any deaths from any cause following the RDA procedure.

## 4 DISCUSSION

An ECG ST elevation, a defining feature of transmural ischemia (damage affecting the complete thickness of the ventricle wall), results from this. By emphasizing variations in the resting membrane potential and action potentials across ischemic and nonischemic zones, depolarization theory offers a crucial explanation for ST elevation. Abnormalities in depolarization interfere with the myocardium's normal electrical impulse conduction, increasing the risk of arrhythmia and causing conduction delays. In patients with BrS, the arrhythmogenic substrate is characterized by highly localized epicardial abnormalities rather than diffuse repolarization changes. Sarcon et al.^24^ demonstrated that, even in the absence of a spontaneous type‐1 ECG pattern, high‐density epicardial mapping consistently identifies regions of low‐voltage, fractionated electrograms, and late potentials confined to the anterior RVOT. These abnormal electrograms correlate directly with the coved‐type ST‐segment elevation, fragmented QRS complexes, and isolated late potentials observed on surface ECG. Moreover, these signals intensify with faster pacing and expand significantly after procainamide infusion, unmasking the Brugada phenotype.^25^ Targeted radiofrequency ablation of these fractionated sites not only abolishes the late potentials and normalizes the ST segment but also prevents recurrent ventricular fibrillation, underscoring a depolarization‐driven mechanism that is uniquely manifest in BrS. Intraventricular conduction delay (IVCD), which is associated with heart failure and sudden cardiac death, and bundle branch block (BBB), which lengthens the QRS duration and results in ventricular dyssynchrony, are important symptoms. Ventricular arrhythmias are linked to fragmented QRS (fQRS), a sign of myocardial fibrosis, especially in ischemia and hypertrophic cardiomyopathies. Abnormalities in repolarization impact myocardial recovery and raise the risk of arrhythmias such as sudden cardiac death and torsades de pointes. While early repolarization syndrome (ERS), characterized by elevated J‐point, is associated with ventricular fibrillation, particularly in young people, a prolonged QT interval implies delayed repolarization, which is frequently caused by genetic mutations or drugs. In structural heart illness, T‐wave alternans (TWA) predicts sudden cardiac mortality and represents repolarization instability. Furthermore, ischemic or inflammatory heart disorders such as acute coronary syndromes, pericarditis, and myocarditis are indicated by ST‐segment deviations.^26^

The findings of this study underscore the therapeutic value of RFA in BrS, particularly in its ability to significantly reduce VA recurrence and ICD shocks. The reduction in VA recurrence and ICD shock rates following RFA underscores its clinical efficacy in managing Brugada syndrome (BrS)‐related arrhythmias. By effectively suppressing arrhythmic activity, RFA provides long‐term rhythm stability, suggesting that the procedure interrupts the abnormal electrical pathways responsible for life‐threatening events, which is essential for enhancing patient survival. Additionally, the decrease in ICD shocks highlights the procedure's role in reducing the frequency of device interventions, lessening patient distress and improving quality of life. With fewer shocks, RFA supports a more stable cardiac rhythm that minimizes ICD activation, offering significant benefits to patient comfort and psychological well‐being. This dual impact on both VA recurrence and ICD shock rates solidifies RFA as an effective strategy for promoting sustained rhythm control and improving overall outcomes in BrS management.^27^

These outcomes also likely stem from the ablation's direct targeting of the arrhythmogenic substrate in BrS, which is predominantly located in the right ventricular epicardium. The abnormal sodium channel function in BrS results in a transmural voltage gradient, leading to ST‐segment elevation and an increased susceptibility to VA. By ablating these epicardial regions, RFA disrupts the conduction pathways that foster reentrant arrhythmias, thereby reducing the incidence of recurrent VA.^27^ A previous systematic review by Fernandes et al. (2018), which included 11 case series and 11 case reports, demonstrated that epicardial substrate modification appears to be a more effective strategy for preventing VT and VF than endocardial ablation alone. Persistent or recurrent J‐ST elevation was found to be indicative of unsuccessful ablation. For patients with VT/VF and BrS, ablation remains a reasonable therapeutic option.^28^ A more recent systematic review by Kotake et al. (2023), comprising 15 case series and 41 case studies, similarly concluded that catheter ablation is an effective method for eliminating the arrhythmogenic substrate associated with BrS and related arrhythmias.^29^ The novelty of our research is that we carried out a quantitative evaluation using meta‐analysis with a different number of studies. We specifically analyzed the long‐term effects of epicardial radiofrequency ablation and its benefits for recurrent ventricular arrhythmia in BrS.

Unlike conventional ablation techniques, which target the endocardium (tissue inside the heart), epicardial RFA targets the epicardium (tissue outside the heart) to treat specific arrhythmias. When endocardial ablation fails to address the epicardial substrate, high‐volume centers routinely perform epicardial ablation for VT in both ischemic and nonischemic cardiomyopathies.^30^ This mechanism explains the limitation of endocardial ablation success in reducing VA or ICD shocks because it does not directly address the epicardial abnormalities responsible for the arrhythmogenesis. The deeper myocardial layers accessed by endocardial ablation may not contain the critical electrophysiological disturbances seen in BrS, explaining the more modest impact on arrhythmia recurrence and device shocks. But because of the nature of the procedure, pericarditis is the most common complication, with operators frequently administering intrapericardial steroids to reduce its incidence. Other potential complications include damage to the liver or diaphragm, particularly when using the subxiphoid entry approach.^31^ By decreasing the need for ICD interventions, RFA helps mitigate these secondary psychological effects, improving both physical and mental health outcomes for BrS patients. This reduction in ICD shocks can be attributed to the successful modification of the electrical circuits responsible for triggering VAs, further emphasizing RFA's role as an effective adjunctive treatment.

Epicardial ablation is generally reserved for patients who have not responded to endocardial ablation, typically in high‐volume, specialized centers. Cooled irrigation techniques have been developed to prevent catheter tip overheating, ensuring sufficient RFA energy delivery during ablation. Early studies suggest that cooled or irrigated‐tip RFA produces larger epicardial lesions compared to standard 4‐mm RFA. Additionally, epicardial fat appears to be particularly well‐suited for irrigated ablation.^32^ Endocardial ablation is used to target PVCs that cause VF, anatomically from the endocardium when ablation cannot be accomplished on the epicardial side because of the presence of coronary arteries or fat, or when targeting VT. This is the reasoning for endocardial ablation in BrS patients.^19^

**FIGURE 4:**
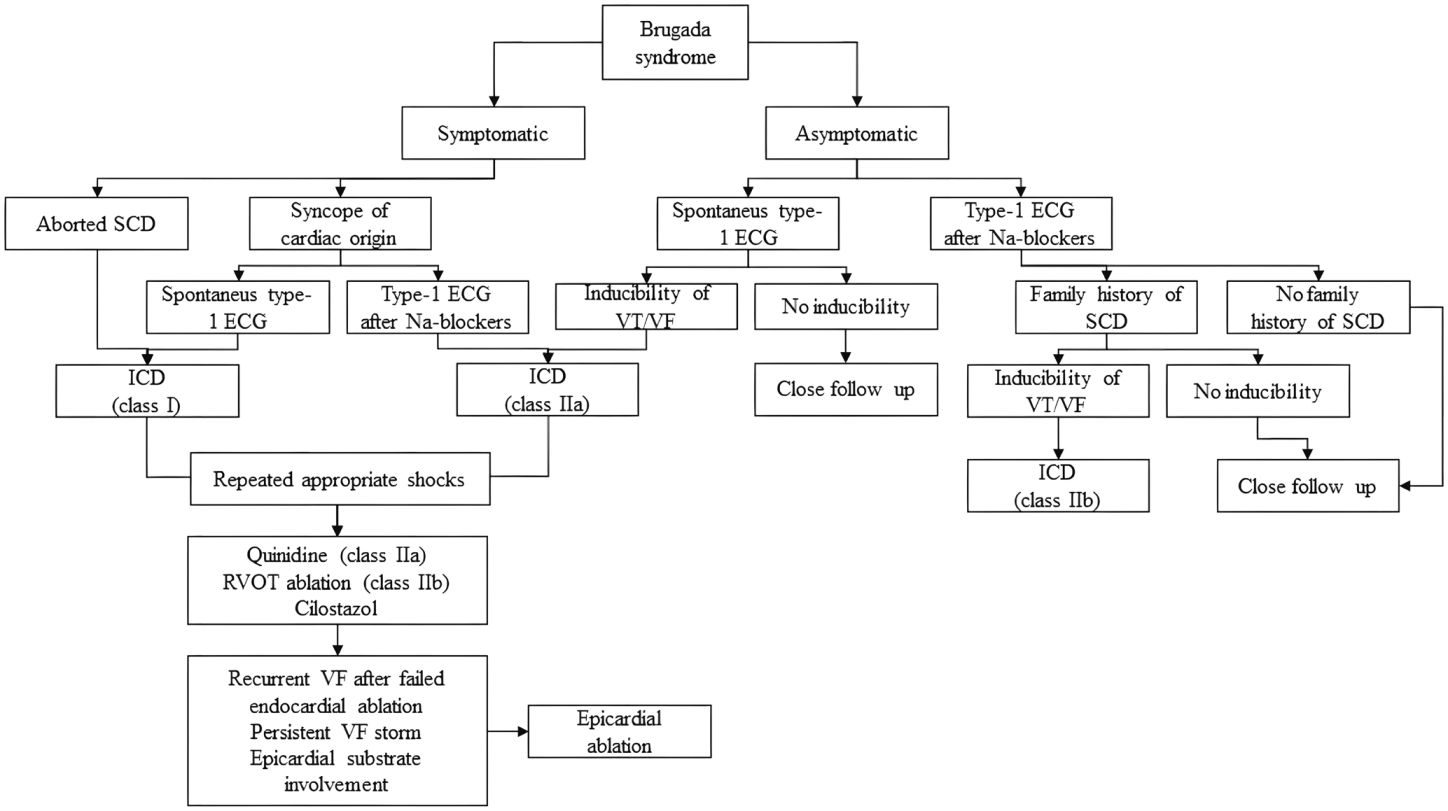
Proposed algorithm for the determination of invasive epicardial ablation, endocardial ablation, and ICD use.^33^

A proposed algorithm for the determination of invasive epicardial ablation, endocardial ablation, and ICD use in BrS patients can be seen in Figure 4. The figure illustrates the management strategy for BrS, a hereditary cardiac disorder that heightens the risk of severe arrhythmias, including VT and VF, which can result in SCD. The management approach is based on whether the patient exhibits symptoms (such as syncope or aborted cardiac arrest) or remains asymptomatic despite having characteristic ECG findings.^33^, ^34^, ^35^

For patients who are symptomatic, particularly those who have survived a SCD episode or experienced unexplained fainting, thorough evaluation is essential. If the ECG displays the typical type 1 Brugada pattern, the implantation of an ICD is highly recommended. This device continuously monitors heart activity and can deliver shocks to terminate life‐threatening arrhythmias. In instances where the type 1 pattern is only identified after administering Na channel blockers, further testing is conducted to assess the inducibility of arrhythmias through an electrophysiology study. If arrhythmias can be induced, ICD implantation is still advised. However, if no arrhythmias are provoked, close monitoring is suggested instead.

In asymptomatic patients, if a spontaneous type 1 ECG pattern is observed, inducibility tests are performed to evaluate arrhythmia risk. Patients showing inducible VT/VF are deemed at higher risk and may benefit from ICD implantation, while those without inducible arrhythmias can be managed through vigilant monitoring. For asymptomatic individuals whose type 1 pattern appears only after Na channel blockers are administered, family history of SCD plays a crucial role in risk assessment. A family history of SCD increases perceived risk, potentially warranting ICD recommendation, especially if VT/VF can be induced. Conversely, in patients without such a history or if no arrhythmias are inducible, close follow‐up without immediate intervention is appropriate.^36^, ^37^

For patients with an ICD who experience recurrent appropriate shocks because of ongoing arrhythmias, additional treatment options may be explored. Medications like Quinidine and procedures such as right ventricular outflow tract (RVOT) ablation may be utilized to manage arrhythmias effectively. In severe cases where VF persists, especially when endocardial ablation fails or when arrhythmias affect the epicardial substrate, an epicardial ablation procedure may be conducted to target and eliminate the problematic areas of the heart.

Based on this study, the inability to locate an RFA target and closeness to a coronary artery or the left phrenic nerve are the primary limitations of epicardial RFA. Epicardial access, mapping, and ablation can result in a special set of consequences, such as pericarditis, significant coronary artery damage, phrenic nerve injury, and hemopericardium. Patient safety depends on anticipating, avoiding, and, if required, addressing these issues. Delivery of epicardial RFA is restricted by fat or linked to phrenic nerve injury, coronary artery damage, hemorrhage, and extra‐cardiac damage.

### 4.1 Study limitations

There is a limited number of studies directly comparing the risks and benefits between RFA and non‐RFA groups. Most available literature consists of case series or case reports, leading to potential biases and limited generalizability. We also observed a high degree of heterogeneity (*I* ^2^ >80%) among the included studies. This variability may be attributed to several factors, such as differences in study design, patient demographics, baseline risk of ventricular arrhythmia, and variations in procedural techniques—including distinctions in operator expertise and follow‐up durations. In particular, geographic and genetic diversity among the study populations, as well as variations in the definitions and measurements of clinical outcomes, likely contributed to the observed heterogeneity. Additionally, research on RFA for BrS is not conducted uniformly across different regions, which may affect the representation of factors related to disease epidemiology and long‐term outcomes. Therefore, further research, particularly well‐designed comparative studies, is required to evaluate the long‐term effects of RFA versus non‐RFA treatments to achieve more comprehensive and globally representative results.

## 5 CONCLUSIONS

From this study, RFA provides significant outcomes for improvement in BrS patients. There was a substantial decrease in the event rates of recurrent VA after epicardial ablation. Moreover, RFA is also related to a substantial decrease in ICD shock in patients. None of the included studies reported any deaths from any cause following the RFA procedure. These findings have important clinical implications for BrS management, suggesting that RFA should be considered a key therapeutic option for the patients. Despite the promising results, the presence of significant heterogeneity across studies raises questions about the variability in procedural techniques, patient selection, follow‐up durations, regional disparities in the study population, and the lack of randomized controlled trials.

## CONFLICT OF INTEREST STATEMENT

The authors have no conflicts of interest to declare.

## PATIENT CONSENT STATEMENT

No human participant was involved in this study.

## Data Availability

The data that support the findings of this study are available from the corresponding author upon reasonable request.
